# Real-time hyperpolarized ^13^C magnetic resonance detects increased pyruvate oxidation in pyruvate dehydrogenase kinase 2/4–double knockout mouse livers

**DOI:** 10.1038/s41598-019-52952-6

**Published:** 2019-11-11

**Authors:** Gaurav Sharma, Cheng-Yang Wu, R. Max Wynn, Wenjun Gui, Craig R. Malloy, A. Dean Sherry, David T. Chuang, Chalermchai Khemtong

**Affiliations:** 1 Advanced Imaging Research Center, University of Texas Southwestern Medical Center, Dallas, TX United States; 2 Department of Biochemistry, University of Texas Southwestern Medical Center, Dallas, TX United States; 3 Department of Internal Medicine, University of Texas Southwestern Medical Center, Dallas, TX United States; 4 Department of Radiology, University of Texas Southwestern Medical Center, Dallas, TX United States; 5 Department of Chemistry, University of Texas at Dallas, Dallas, TX United States

**Keywords:** Biochemistry, Physiology

## Abstract

The pyruvate dehydrogenase complex (PDH) critically regulates carbohydrate metabolism. Phosphorylation of PDH by one of the pyruvate dehydrogenase kinases 1–4 (PDK1–4) decreases the flux of carbohydrates into the TCA cycle. Inhibition of PDKs increases oxidative metabolism of carbohydrates, so targeting PDKs has emerged as an important therapeutic approach to manage various metabolic diseases. Therefore, it is highly desirable to begin to establish imaging tools for noninvasive measurements of PDH flux in rodent models. In this study, we used hyperpolarized (HP) ^13^C-magnetic resonance spectroscopy to study the impact of a PDK2/PDK4 double knockout (DKO) on pyruvate metabolism in perfused livers from lean and diet-induced obese (DIO) mice and validated the HP observations with high-resolution ^13^C-nuclear magnetic resonance (NMR) spectroscopy of tissue extracts and steady-state isotopomer analyses. We observed that PDK-deficient livers produce more HP-bicarbonate from HP-[1-^13^C]pyruvate than age-matched control livers. A steady-state ^13^C-NMR isotopomer analysis of tissue extracts confirmed that flux rates through PDH, as well as pyruvate carboxylase and pyruvate cycling activities, are significantly higher in PDK-deficient livers. Immunoblotting experiments confirmed that HP-bicarbonate production from HP-[1-^13^C]pyruvate parallels decreased phosphorylation of the PDH E1α subunit (pE1α) in liver tissue. Our findings indicate that combining real-time hyperpolarized ^13^C NMR spectroscopy and ^13^C isotopomer analysis provides quantitative insights into intermediary metabolism in PDK-knockout mice. We propose that this method will be useful in assessing metabolic disease states and developing therapies to improve PDH flux.

## Introduction

The pyruvate dehydrogenase (PDH) complex plays a central role in metabolic regulation^1,2^. PDH catalyzes the irreversible oxidative decarboxylation of pyruvate to acetyl-CoA and links glucose metabolism to the tricarboxylic acid (TCA) cycle^3,4^. PDH is regulated by several factors including nutritional state and cellular energy demand as well as concentrations of pyruvate, acetyl-CoA, ATP, and NADH. The activity of PDH is also rapidly regulated by phosphorylation and dephosphorylation by pyruvate dehydrogenase kinase (PDK) and pyruvate dehydrogenase phosphatases (PDP), respectively^5–7^. Prolonged feeding of a diet high in saturated fats increases PDK expression in rodent models^8,9^. Increased PDK expression in obesity contributes to lower PDH activity and reduced oxidation of carbohydrates^10^. This suggests that inhibition of PDK 2 and PDK 4 may reverse adverse metabolic effects by increasing pyruvate oxidation.

A number of studies have shown that targeting PDK activity is indeed promising for the treatment of obesity-related metabolic conditions^11–14^. Small molecules such as dichloroacetate inhibit PDKs and administration is associated with improved glucose tolerance in rodent models^13–16^. However, because dichloroacetate is carcinogenic and relatively nonspecific for PDK, alternative approaches for inhibition of PDK have been developed. For example, Tso *et al*. recently reported that targeting PDK with 2-[(2,4-dihydroxyphenyl)sulfonyl] isoindoline-4,6-diol (designated PS10) resulted in improved glucose tolerance in mice^16^. Genetic knockouts of PDK isoforms have also been reported^11,12,17^. Consistent with results from pharmacological inhibition, Jeoung *et al*. found that knockout of both PDK 2 and 4 (double knockout, DKO) in mice resulted in increased PDH activity and lower blood glucose levels in both fed and fasted states^12^. In addition and somewhat unexpectedly, hepatic steatosis was improved in obese animals with either pharmacological inhibition of PDK 2/4 or knock out of PDK 2/4. Thus, monitoring the effects of PDK inhibition on pyruvate metabolism will be useful for understanding metabolic disorders and developing therapies targeting this system.

These earlier studies relied on invasive analysis of tissue extracts. Noninvasive hyperpolarization methods have been introduced to assess the alternative pathways of pyruvate metabolism in intact tissue with the prospect of human applications^18,19^. Metabolism of [1-^13^C]pyruvate through PDH yields [^13^C]bicarbonate^20^. Flux through PC may also yield [^13^C]bicarbonate through multiple reaction steps^20–22^. After administration of HP [1-^13^C]pyruvate, C1- and C4-labeled aspartate and malate appear as a consequence of flux through pyruvate carboxylase followed by scrambling in the symmetric fumarate and succinate pools^21,23,24^. Consequently, in principle HP [^13^C]bicarbonate may arise through decarboxylation via four enzymes: the malic enzyme, PEPCK, isocitrate dehydrogenase or alpha-ketoglutarate dehydrogenase. Rodent models of type 1 diabetes and obesity are associated with increased expression of pyruvate carboxylase which may indicate that a significant source of HP [^13^C]bicarbonate may reflect pyruvate carboxylation^25^. Sources of [^13^C]bicarbonate in the liver in models of obesity and the sensitivity to changes in PDK activity have not been evaluated, and results in the literature are conflicting. In a study of lean rats using hyperpolarization methods and validation with conventional ^13^C methods, HP [^13^C]bicarbonate production was detected in fed but not fasted animals^22,26^. In contrast, in a study of diet-induced obesity in the mouse, where PDH activity is thought to be reduced, even with 24 hours of fasting the appearance of [^13^C]bicarbonate was attributed to flux through PDH^23^. In isolated livers supplied with fatty acids which suppressed flux in PDH, much of the [^13^C]bicarbonate production was via PC and downstream pathways^20,21^.

It is important to understand the relative contributions of PDH and PC flux to bicarbonate production to assess interventions targeting PDKs *in vivo* using HP methods. The current study was designed to determine whether the appearance of [^13^C]bicarbonate after administration of [1-^13^C]pyruvate can be used as a reliable indicator of PDH flux in diet-induced obesity. Non-polarized ^13^C-enriched substrates were also present during the HP experiment, but these metabolites were undetectable on the time-scale of the HP exam. Coupled with measurements of hepatic oxygen consumption, flux through PDH versus PC could be calculated in livers from PDK2/PDK4 double knockout (DKO) mice exposed to a normal or high-fat diet. The correlation between the appearance of HP ^13^C-bicarbonate and the knockout of hepatic PDK enzymes is important for translating HP ^13^C-MRS as a noninvasive imaging tool for the treatment and management of chronic liver diseases.

## Results

### Real-time ^13^C magnetic resonance spectroscopy detects increased production of hyperpolarized bicarbonate in PDK-deficient livers

**Figure 1:**
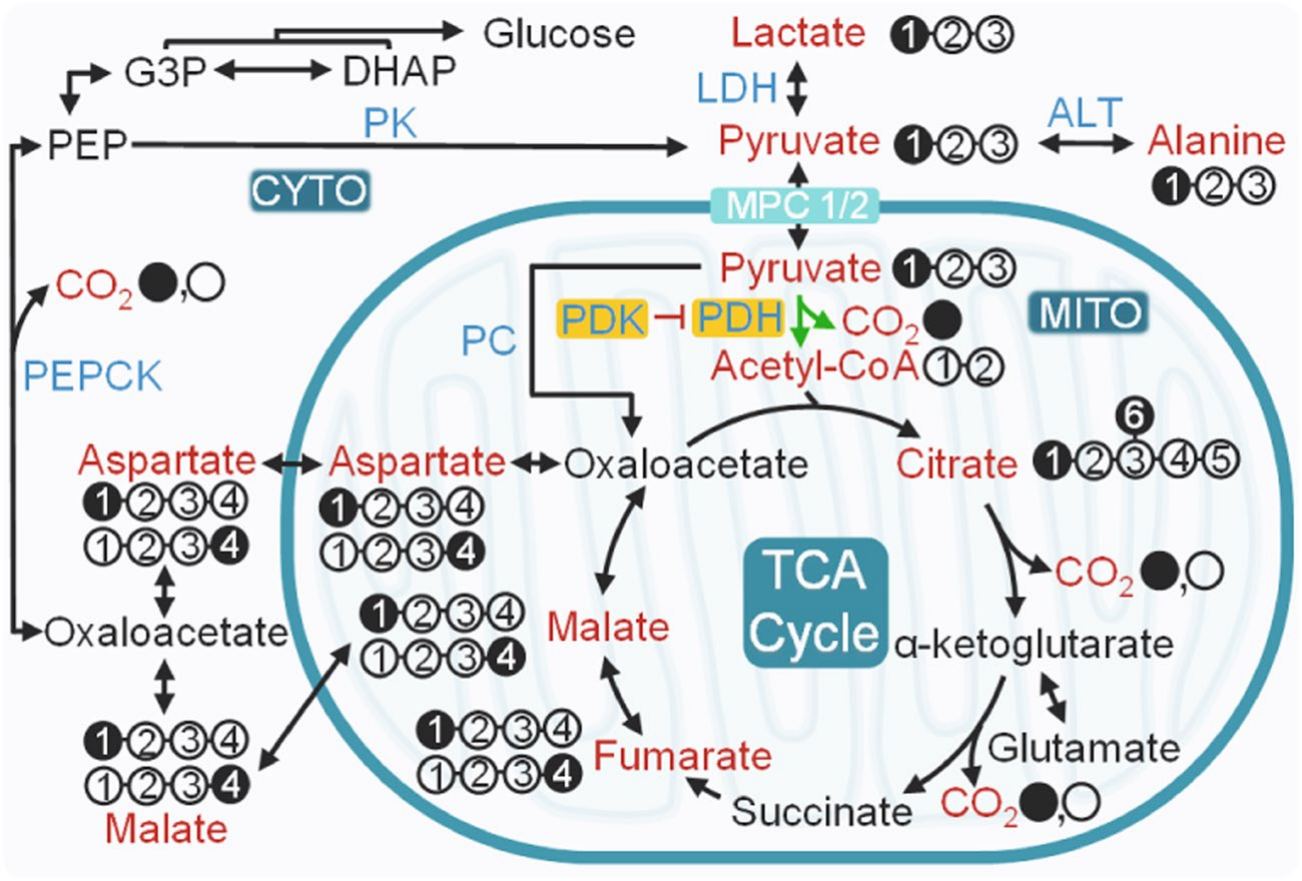
Metabolic fates of HP [1-^13^C]pyruvate in an isolated perfused mouse liver. Filled circles represent ^13^C-enriched carbons while the open circles denote carbon atoms without ^13^C-enrichment. Metabolites labeled with HP ^13^C isotope from HP [1-^13^C]pyruvate, therefore potentially traceable by ^13^C NMR, are shown in red. All four-carbon intermediates are shown as two isotopomers with ^13^C-labelling at either the C1 or the C4 position. The intermediates with ^13^C-labelling at C1 are produced by direction carboxylation of HP [1-^13^C]pyruvate to [1-^13^C]oxaloacetate. Metabolism of the resulting [1-^13^C]oxaloacetate to [1-^13^C]malate followed by backward scrambling by fumarase results in the production of four-carbon intermediates with ^13^C-labelling at C4. ALT: alanine transaminase; CYTO: cytosol; G3P: glyceraldehyde 3-phosphate; LDH: lactate dehydrogenase; MITO: mitochondria; MPC1/2: mitochondrial pyruvate carrier 1 and 2; PDH: pyruvate dehydrogenase complex; PDK: pyruvate dehydrogenase kinase; PC: pyruvate carboxylase; PEP: phosphoenolpyruvate; PEPCK: phosphoenolpyruvate carboxykinase and TCA: tricarboxylic acid.

**Figure 2:**
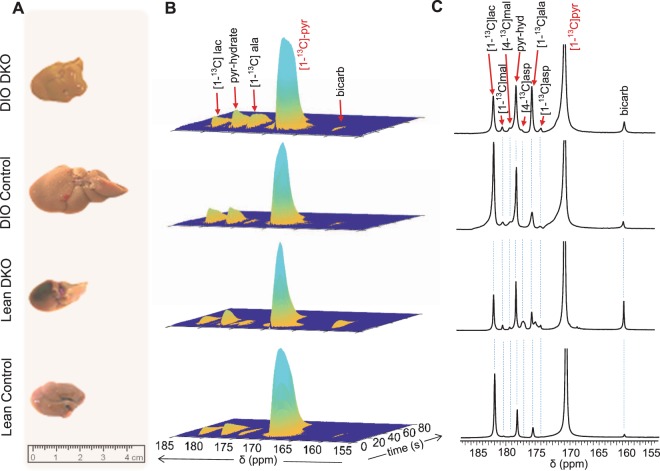
Time-resolved HP ^13^C MR of isolated perfused livers after giving HP [1-^13^C]pyruvate. (**A**) Representative photographs of the isolated livers from all four groups of mice used in this study; (**B**) time-resolved ^13^C NMR signals of perfused mouse livers after receiving HP [1-^13^C]pyruvate (2 mM); and (**C**) representative ^13^C NMR spectra of the perfused livers obtained by summing 50 free-induction decays acquired over 100 s. Compared to their respective controls, ^13^C bicarbonate was increased in the double knockouts, consistent with increased flux through pyruvate dehydrogenase.

The potential pathways for metabolism of HP [1-^13^C]pyruvate in a liver are illustrated in Fig. 1. Livers isolated from the four groups of mice varied in size, with DIO control livers being significantly larger (Fig. 2A) than those from other groups reflective of fat accumulation^11,16^. The average weights of the isolated livers were 1.51 ± 0.28 g, 1.58 ± 0.46 g, 3.81 ± 0.44 g, and 2.10 ± 0.58 g for lean control, lean DKO, DIO control, and DIO DKO mice, respectively (Fig. S1A). During the HP ^13^C NMR examination, multiple metabolic products of pyruvate were detected in all groups of livers shortly after the injection of HP [1-^13^C]pyruvate (Fig. 2B). Representative summed ^13^C spectra (50 spectra collected over 100 s) are displayed in Fig. 2C. ^13^C resonances reflecting [1-^13^C]pyruvate, [^13^C]bicarbonate (160.9 ppm), [1-^13^C]aspartate (175.3), [1-^13^C]alanine (176.5 ppm), [4-^13^C]aspartate (178.3), [4-^13^C1]malate (180.3 ppm), [1-^13^C4]malate (181.5 ppm) and [1-^13^C]lactate (183.1 ppm) were all visible. These results are consistent with previous reports on the metabolism of HP [1-^13^C]pyruvate via both PC and PDH.

**Figure 3:**
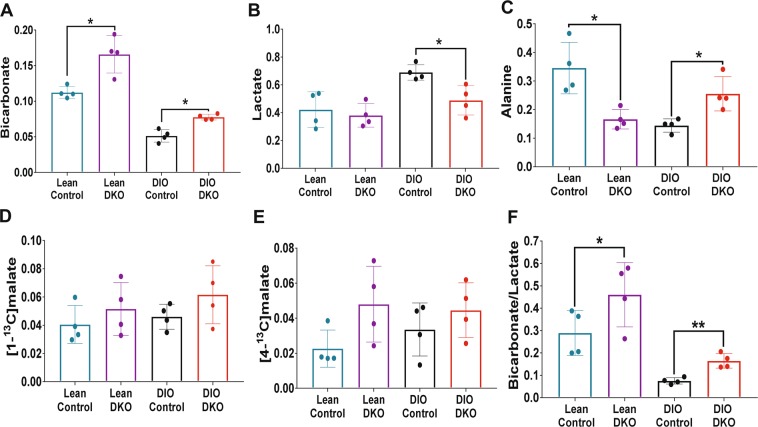
The impact of PDK DKO on HP [1-^13^C]pyruvate metabolism. Normalized fractional intensities of (**A**) bicarbonate, (**B**) lactate, and (**C**) alanine; (**D**) [1-^13^C]malate, (**E**) [4-^13^C]malate, and (**F**) bicarbonate/lactate ratio; Data presented as the mean ± SD (n = 4 per group) with statistical significance of differences indicated by “*” (*P* < 0.05) and “**” (*P* < 0.001). DIO: diet-induced obese; and DKO: double knockout of PDK2/4.

The resonances of lactate and alanine, dominant in all spectra, reflect rapid exchange with HP-pyruvate through single enzyme-catalyzed steps, lactate dehydrogenase, and alanine aminotransferase, respectively. A larger ^13^C-bicarbonate signal was observed in DKO livers from both lean and obese animals with the lean DKO livers producing the most ^13^C-bicarbonate (Fig. 2B,C). The average signal intensities of ^13^C-bicarbonate, [1-^13^C]lactate, and [1-^13^C]alanine normalized to the total signal of all HP ^13^C metabolites are shown in Fig. 3A–C, respectively. From these plots, it is clear that more HP ^13^C-bicarbonate was generated from HP-pyruvate in livers from lean DKO mice than those from the wild-type groups in both lean and obese animals (P < 0.05). It was also evident that less HP ^13^C-bicarbonate was produced in obese livers compared to the lean groups.

The amount of HP [1-^13^C]lactate produced in these livers (Fig. 3B) showed the opposite trend. Here, more HP-[1-^13^C]lactate was observed in livers from DIO obese animals likely reflecting a more highly reduced tissue redox state. The amount of HP-lactate was lower in DIO DKO animals compare to DIO controls, perhaps reflecting greater flux of HP-pyruvate through PDH and less through LDH. This was less evident in the lean controls versus lean DKO comparisons. A somewhat different pattern was observed in the appearance of HP [1-^13^C]alanine (Fig. 3C). In the lean groups, less HP [1-^13^C]alanine was found in DKO livers compared to controls (P < 0.05) yet there was more HP-[1-^13^C]alanine detected in obese DIO DKO livers compared to DIO controls, the livers that accumulated the most fat (P < 0.05).

The signals of the 4-carbon TCA cycle intermediates, ^13^C-malate and ^13^C-aspartate, reflects carboxylation of HP [1-^13^C]pyruvate to form oxaloacetate. The NMR signals of [1-^13^C]malate and [4-^13^C]malate (Fig. 3D,E) tended to be more intense in DKO livers compared to controls but these differences did not reach statistical significance. Also, the intensity of the [1-^13^C]malate resonances tended to be higher than the intensity of the [4-^13^C]malate resonances. This reflects incomplete scrambling of [1-^13^C]oxaloacetate (formed directly by carboxylation of [1-^13^C]pyruvate) through fumarase to form equivalent amounts of [4-^13^C]oxaloacetate, [4-^13^C]aspartate, and [4-^13^C]malate.

Finally, the ratio of HP-bicarbonate to HP-lactate has previously been used as an overall index for the metabolism of HP [1-^13^C]pyruvate *in vivo*. These ratio comparisons are shown in Fig. 3F. This index was higher in both lean groups (control and DKO) than in both DIO groups (control and DKO). The index was significantly higher in both DKO groups compared to their respective controls. Moreover, a higher bicarbonate/lactate ratio was observed in DIO DKO livers compared to DIO control livers.

### Alternative pathways of pyruvate metabolism and bicarbonate production from isotopomer analysis

**Figure 4:**
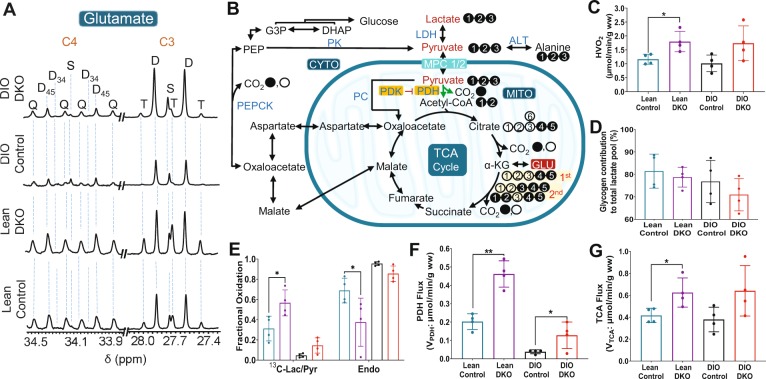
Metabolic parameters obtained by ^13^C NMR isotopomer analysis of tissue glutamate. (**A**) glutamate C-4 (34.20 ppm) and C-3 (27.60 ppm) resonances; (**B**) a metabolic scheme showing ^13^C-labeling of glutamate isotopomers; (**C**) oxygen consumption; (**D**) contribution of glycogen to the total lactate pool; (**E**) fractional oxidation of ^13^C-lactate/^13^C-pyruvate and endogenous sources for energy production by the TCA cycle; (**F**) pyruvate dehydrogenase (PDH) flux; and (**G**) tricarboxylic acid cycle (TCA) flux. The letters S, D, T, and Q refer to a singlet, doublet (with the relevant *J*-coupled spins), triplet (a degenerate doublet of doublets), and quartet (or doublet of doublets), respectively. Filled circles in the metabolic scheme represent ^13^C-enriched carbons while open circles represent natural abundance levels of ^13^C. ^13^C-enriched pyruvate and lactate supplied in the perfusion medium are shown in red in (**B**). For viewing simplicity, only ^13^C-labeled glutamate isotopomers generated after the 1^st^ and 2^nd^ turns are shown. ^13^C-labeling patterns of other metabolites can be generated using tcaSIM and tcaCALC (downloadable free of charge at http://www.invivometabolism.org/tca.html. Abbreviations: α-KG = α-ketoglutarate; ALT = alanine transaminase; CYTO = cytosol; G3P = glyceraldehyde 3-phosphate; GLU = glutamate; LDH = lactate dehydrogenase; MITO = mitochondria; MPC1/2 = mitochondrial pyruvate carrier 1 and 2; PDH = pyruvate dehydrogenase complex; PDK = pyruvate dehydrogenase kinase; PC = pyruvate carboxylase; PEP = phosphoenolpyruvate; PEPCK = phosphoenolpyruvate carboxykinase, TCA = tricarboxylic acid; and gww = gram wet weight. The bar plots represent the mean ± SD (n = 4 per group) with significance indicated by “*” (*P* < 0.05) and “**” (*P* < 0.001).

The temporal resolution for the appearance of [^13^C]bicarbonate after pyruvate administration was on the order of a few seconds (Fig. 2), but the metabolic pathways leading to bicarbonate production cannot be determined from hyperpolarization data. For this reason, the same livers were also perfused with medium containing 1.5 mM [U-^13^C]lactate and 0.15 mM [U-^13^C]pyruvate before and after the HP experiment, followed by tissue extraction and analysis that is independent of hyperpolarization results. Examples of the ^13^C NMR spectrum of glutamate C3 and C4 for each of the four groups of livers are compared in Fig. 4. Oxidation of [U-^13^C]pyruvate produces [1,2-^13^C_2_]acetyl-CoA, which enters the TCA cycle to produce [4,5-^13^C_2_]α-ketoglutarate. The ^13^C-labeled metabolic intermediate rapidly exchanges with glutamate, resulting in glutamate with ^13^C-labeling at C4 and C5 positions ([4,5-^13^C_2_]glutamate). This labeling pattern appears as a doublet (D_45_) in C4 multiplet. Further metabolism of [4,5-^13^C_2_]α-ketoglutarate through the TCA cycle produces [1,2-^13^C_2_]oxaloacetate or [3,4-^13^C_2_]oxaloacetate following hydration of fumarate (fumarase). Subsequently, these oxaloacetate condense with newly produced [1,2-^13^C_2_]acetyl-CoA, eventually producing [3,4,5-^13^C_3_]glutamate and [1,2,4,5-^13^C_4_]glutamate. The C4 carbon resonance of [3,4,5-^13^C_3_]glutamate appears as a doublet of doublets or quartet (Q). The larger doublet of doublets (or quartet) in glutamate C4 and the larger doublet of doublets (or triplet) in glutamate C3 were consistent with higher fractional ^13^C enrichment in acetyl-CoA in lean animals. The scheme in Fig. 4B explains the appearance of ^13^C-multiplets as a result of [U-^13^C]pyruvate metabolism.

Hepatic oxygen consumption (Fig. 4C) was significantly higher in livers from lean DKO animals (1.79 ± 0.36 µmol/min/gww) compared to livers from lean controls (1.16 ± 0.18 µmol/min/gww). However, an increase in oxygen consumption was also observed in DIO DKO (1.73 ± 0.62 µmol/min/gww) compared to DIO controls (1.01 ± 0.29 µmol/min/gww), but these differences did not reach statistical significance. The DKO of PDK2/4 renders the liver metabolically more active but the subsequent effects of high-fat diet such as hepatic steatosis/fat accumulation in tissue and weight gain^27^ (Fig. S1B) were also observed in the DIO DKO livers. In all groups, unlabeled acetyl-CoA was detected in spite of providing only ^13^C labeled pyruvate and lactate in the medium. The unlabeled material must arise from either glycogen or endogenous triglycerides. A comparison of glycogen contribution to the total pyruvate and lactate pool in these livers is shown in Fig. 4D. A small decrease was observed in glycogen contribution from lean control (81.40 ± 7.59%) to DIO control (76.80 ± 9.34%) and from lean DKO (78.72 ± 4.42%) to DIO DKO (70.93 ± 7.17%). The fractional contribution of unlabeled sources is shown in Fig. 4E, demonstrating higher unlabeled contribution to acetyl-CoA in DIO animals.

PDH flux (V_*PDH*_, Fig. 4F) estimated from ^13^C isotopomer analysis of glutamate multiplets (see Fig. S2) was significantly higher in livers from lean DKO animals (0.46 ± 0.07 µmol/min/gww) and DIO DKO animals (0.12 ± 0.07 µmol/min/gww) than their respective controls (Lean control: 0.20 ± 0.04 µmol/min/gww and DIO Control: 0.03 ± 0.01 µmol/min/gww). A similar trend was also observed for TCA cycle flux (V_*TCA*_, Fig. 4G). The flux values for lean DKO, DIO DKO, lean control, and DIO control was 0.62 ± 0.13, 0.64 ± 0.23, 0.41 ± 0.06, and 0.37 ± 0.11 µmol/min/gww, respectively. Thus, liver metabolism with predominant fat oxidation (DIO controls) switches to a metabolic state in which increased carbohydrate contribution as an energy source is achieved (DIO DKO) by increasing both V_*PDH*_ and V_*TCA*_.

**Figure 5:**
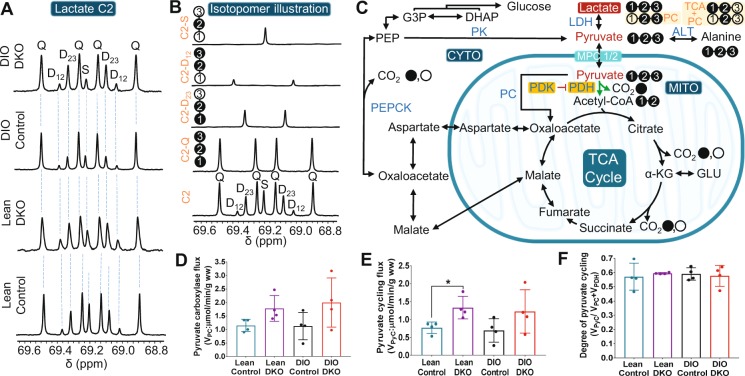
Metabolic parameters obtained by ^13^C NMR isotopomer analyses of lactate and glutamate in tissue extracts. (**A**) multiplets in lactate C-2 (69.20 ppm); (**B**) simulated spectra showing singlet, doublet, and quartet and sum of multiplets in lactate C-2; (**C**) a scheme illustrating the production of all possible ^13^C-labeled lactate isotopomers; (**D**) pyruvate carboxylase (V_*PC*_) flux; (**E**) pyruvate cycling flux (V_*PK*_); and (**F**) degree of pyruvate cycling in each group of livers. The letters S, D, T, and Q refer to a singlet, doublet (with the relevant J-coupled spins), triplet (a degenerate doublet of doublets), and quartet (doublet of doublets), respectively. Filled and open circles in the metabolic scheme represent ^13^C-enriched and natural abundant ^13^C carbons in lactate, respectively. The remaining abbreviations are identical to those given in Fig. 4. The bar plots reflect the mean ± SD (n = 4 per group) with a statistical significance of differences indicated by “*” (*P* < 0.05).

Pyruvate carboxylase flux (V_*PC*_), however, was not different among these groups. V_*PC*_ for lean control, lean DKO, DIO control, and DIO DKO was 1.14 ± 0.23, 1.78 ± 0.47, 1.12 ± 0.50, and 1.99 ± 0.90 μmol/min/gww, respectively (Fig. 5D). The trend suggests that higher PC flux in DKO livers in both lean and DIO groups. However, the increase was small and the difference was not statistically significant. In summary, knockout of PDKs was associated with significantly increased PDH flux in both lean and DIO animals and no significant increase in PC flux. Together, these results suggest that the increased appearance of [^13^C]bicarbonate by HP is a useful biomarker of PDH flux in mouse models of obesity.

### Pyruvate cycling was observed in all perfused livers

In this study, the only ^13^C-labeled compounds were [U-^13^C]lactate and [U-^13^C]pyruvate. Pyruvate cycling was defined here as any pathway involving carboxylation of pyruvate followed by decarboxylation of another TCA cycle intermediate that ultimately regenerates pyruvate. Flux through the pathway pyruvate » oxaloacetate » malate » pyruvate (where malic enzyme is the key step) and flux through the pathway pyruvate » oxaloacetate » PEP » pyruvate (where pyruvate kinase is the key step) are both included. If pyruvate cycling is active, it can be detected in the ^13^C NMR spectrum of both lactate and glutamate.

The ^13^C NMR spectrum of lactate C2 is shown in Fig. 5A in all groups, a doublet due to J12 and a doublet due to J23 was observed in the ^13^C NMR spectrum. The doublets due to J12 and J23 in C2 of lactate (Fig. 5A) can only arise from flux of 4-carbon intermediates from the TCA cycle and back into pyruvate and lactate via pyruvate kinase (PK) or an equivalent pathway (Fig. 5B). There was no difference in the contribution of these doublets to the lactate signal among the four groups. As illustrated in Fig. 5B, the regeneration of pyruvate from the ^13^C-enriched 4-carbon intermediates results in pyruvate and lactate molecules with various ^13^C-labeling patterns. Metabolism of [U-^13^C]pyruvate through PC generates two different OAA isotopomers: [1,2,3-^13^C_3_]- and [2,3,4-^13^C_3_]OAA, and subsequent pyruvate cycling results in [2,3-^13^C_2_]lactate. While metabolism of [U-^13^C]pyruvate after the first TCA cycle turn followed by pyruvate cycling produces [1,2-^13^C_2_]lactate and [3-^13^C]lactate. [1-^13^C]lactate and [2-^13^C]lactate has produced from pyruvate cycling of 4-carbon intermediates generated after [U-^13^C]pyruvate has completed the second turn of the TCA cycle. Because the lactate ^13^C signal also includes lactate in the medium, further quantitative analysis was not performed.

The ^13^C NMR spectrum of glutamate is also sensitive to pyruvate cycling. Results for pyruvate carboxylation were described above and are shown in Fig. 5D. Pyruvate cycling flux (V_*PyC*_) correlates well with the changes in V_*PC*_ (Fig. 5E). DKO livers have higher V_*PyC*_ (1.33 ± 0.31 μmol/min/gww for lean DKO and 1.22 ± 0.60 μmol/min/gww for DIO DKO) while lower V_*PyC*_ values were found for wild-type control livers (0.76 ± 0.16 μmol/min/gww for lean control and 0.69 ± 0.32 μmol/min/gww for DIO control). The degree of hepatic pyruvate cycling as estimated from V_*PyC*_/(V_*PC*_ + V_*PDH*_)^28,29^ was 0.57 ± 0.09, 0.59 ± 0.01, 0.58 ± 0.04, and 0.57 ± 0.07 for lean control, lean DKO, DIO control, and DIO DKO, respectively (Fig. 5F). These results indicate that pyruvate cycling expressed as a fraction of TCA cycle flux is not sensitive to either DIO or DKO status. These results are consistent with the similarity of the ^13^C NMR spectra of lactate across the 4 groups (Fig. 5A).

### PDK deficiency results in reduced fat accumulation

**Figure 6:**
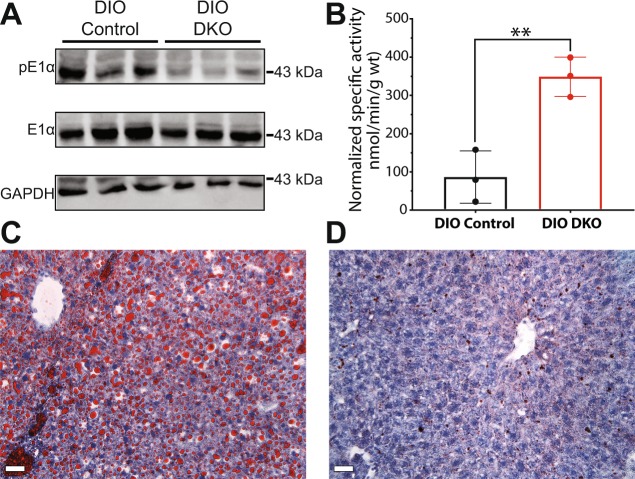
Western blots, specific activity, and histology. (**A**) Western blots showing phosphorylated PDH E1α subunit (pE1α), total phosphorylated PDH E1α subunit in tissue lysates from DIO control and DIO DKO livers (n = 3 livers). GAPDH serves as a loading control. The images were collected from three gels. Raw images of these three gels are shown in Supplementary Information (Fig. S5). (**B**) PDH specific activity; Representative Red Oil O stains from (**C**) DIO control and (D) DIO DKO livers. The red color in each image is attributed to stained lipids within the liver. The scale bars are 20 µm. The bar graph in (**B**) reflects the mean ± SD (n = 3 per group) with a statistical significance of differences indicated by “*” (*P* < 0.05) and “**” (*P* < 0.001).

Phosphorylation of Ser293 on the E1α subunit of PDH by one of the PDKs results in inhibition of the activity of PDH. Western blot analyses of tissue samples (Fig. 6A) indeed showed that phosphorylation of the E1α subunit was very low in DIO DKO livers compared to DIO control livers. Furthermore, the activity of PDH measured in tissue homogenate samples showed that PDH activity was more than 3-fold higher in DIO DKO livers compared to DIO controls (Fig. 6B). The two-fold increase of PDH activity in liver (Fig. 6B) is similar to changes in the appearance of HP [^13^C]bicarbonate (Fig. 2B) in DIO DKO livers. DIO control livers (Fig. 6C) showed significant accumulation of fat while livers from the DKO mice showed minimal fat accumulation (Fig. 6D). To further validate the effect of PDK deficiency in livers, we analyzed glucose production in a representative set of lean control and lean DKO mice. ^13^C spectra of glucose converted to monoacetone glucose (MAG)^30^ indicated that the lean-DKO mice have profoundly lower gluconeogenesis compared to lean control (Fig. S3). These results support earlier reports that double knockout of PDK2/PDK4 restores hepatic metabolism of glucose in obese/pre-diabetic states.

## Discussion

PDH kinases modulate the metabolic state of the liver and consequently have become key drug targets for metabolic diseases such as type 2 diabetes and hepatic steatosis. Tao *et*. *al*. reported that the deficiency of both PDK2 and PDK4 (PDK2/4^−/−^) improves insulin sensitivity in DKO mice^31^. Similarly, Jeoung *et*. *al*. showed that inactivation of PDK2/4 in mice resulted in hypoglycemia and ketoacidosis^12^. This same study also suggested that these two PDK isoforms are sensitive to the nutrition states, with increased PDK2 sensitivity in the fed state and increased PDK4 sensitivity observed in the fasted state. The sensitivity of these two PDK isoforms to nutritional states was corroborated by higher levels of PDK2 and PDK4 expressions in the fed and fasted state, respectively^32^. Both PDK2 and PDK4 isoforms are attractive metabolic targets for pharmacological interventions to treat diabetes and obesity. Real-time evaluation of substrate metabolism as a result of double knockouts of these enzymes are therefore highly desirable.

The increased production of HP-bicarbonate from HP-[1-^13^C]pyruvate in livers from PDK knockout animals was readily detected by ^13^C NMR spectroscopy. Previous studies by Go *et al*. reported increased flux through PDH in PDK4 knockout livers using LCMS^33^. Hyperpolarized [^13^C]bicarbonate resulted from the decarboxylation of [1-^13^C]pyruvate has been used as an indicator of PDH flux^26,34,35^. Jin *et*. *al*. demonstrated that the detection of HP ^13^C-bicarbonate appearance from HP-[1-^13^C]pyruvate in healthy rat livers indicated the PDH activity rather than phosphoenolpyruvate carboxykinase (PEPCK) or TCA cycle activity after pyruvate carboxylation^26^. Similarly, Cunningham *et*. *al*. showed the production of HP-^13^C-bicarbonate from HP-[1-^13^C]pyruvate for *in vivo* assessment of PDH flux in human myocardium^36^. Our results show increased HP ^13^C-bicarbonate signals in double PDK knockout livers, thereby confirming the sensitivity of HP ^13^C-bicarbonate appearance to the genetic knockout. In obese livers, knockout of PDK restored the PDH activity to a certain degree as shown by an increase in HP ^13^C-bicarbonate. However, neither *V*_*PDH*_ nor HP ^13^C-bicarbonate was fully restored to the levels of lean DKO livers. The inability of PDK2/4 knockout to fully dephosphorylate and augment PDH activity in livers likely reflects the presence of other active PDK isoforms, for example, PDK1^37^. It is important to point out that production of HP ^13^C-malate was also higher in DKO livers. This shows directly that there was an increase in flux HP ^13^C-pyruvate into TCA cycle intermediates via PC in PDK-deficient livers.

The Western blot and histology studies of DKO mice fed with high-fat diet confirmed the metabolic findings of NMR. These results reflect modulated PDH activity by reversible phosphorylation at Ser293 on the E1α subunit. The decreased phosphorylation of the E1α subunit (pE1) in the liver homogenate correlates well with the increase in production of HP-bicarbonate and PDH activity in the liver. As a result of increased PDH and anaplerosis fluxes, overall glucose metabolism was elevated in the liver resulting in suppression of liver steatosis via the *de novo* lipogenesis pathway^38^. It has also been recently reported that lower blood glucose levels in HFD-fed DKO mice suppress carbohydrate responsive element binding protein (ChREBP)-mediated lipogenesis^39^. PDK2/PDK4 double knockout is also correlated with increased energy expenditure in the HFD-fed mice. These results suggest that targeting activation of PDH is therapeutically beneficial to obese or Type 2 diabetic patients.

Taken together, our results demonstrate that the metabolism of pyruvate plays an important role in the diagnosis and prognosis of metabolic disorders. Detection of elevated hyperpolarized [^13^C] bicarbonate in PDK deficient livers will facilitate assessing the PDK activity in metabolic complications and determine the efficacy of new therapies targeting PDKs. We have demonstrated that MRS coupled with hyperpolarized ^13^C-labeled pyruvate is capable of revealing the activity of critical enzymes involving energy homeostasis, as well as assessing the PDH and associated fluxes of interest in typical hepatic metabolic diseases such as type 2-diabetes, hepatic steatosis, cirrhosis, and nonalcoholic fatty liver disease. It is noteworthy that detection by hyperpolarized metabolic products by NMR is rapid, noninvasive, and radiation-free and hence is entirely appropriate for translation to human patients.

## Methods

### Animal model and liver perfusion

The animal study was approved by the Institutional Animal Care and Use Committee (IACUC) at UT Southwestern Medical Center. All animal procedures were performed in accordance with IACUC’s guidelines and regulations. Metabolism of HP [1-^13^C] pyruvate was investigated in livers from wild-type and DKO mice aged 6-8 weeks (C57BL/6 J, n = 4 per group). Four groups of mice were studied: 1. Lean control, 2. Lean DKO, 3. diet-induced obese (DIO) control, and 4. DIO DKO. Mice from DIO groups were fed *ad libitum* with a 60% high-fat diet containing 32% saturated and 68% unsaturated fat (Research Diet Inc., New Brunswick, NJ) for 18 weeks while the mice from lean groups were fed a standard chow diet.

Livers were catheterized, isolated, and perfused as previously described^24,40^. Briefly, the liver and hepatic circulatory system was exposed through a midline-laparotomy, the hepatic portal vein was cannulated, dissected and simultaneously perfused using a modified Krebs-Henseleit (KH) solution (25 mM NaHCO_3_, 118 mM NaCl, 4.7 mM KCl, 1.2 mM MgSO_4_, 1.2 mM KH_2_PO_4_ and 1.25 mM CaCl_2_) containing 1.5 mM [U-^13^C]lactate and 0.15 mM [U-^13^C]pyruvate at a constant pressure of 25 cm-H_2_O. The perfusion buffer was oxygenated with a 95:5 mixture of O_2_/CO_2_. The perfusion timeline is shown in Fig. S1B. Oxygen consumption was calculated as described previously^41^ using efferent and afferent *p*O_2_ measurements made with a blood gas analyzer (Instrumentation Laboratory, Lexington, MA, U.S.A.). Prior to ^13^C NMR, shimming was carried out on the ^23^Na FID to obtain the linewidth of ~15 Hz, while the liver was surrounded by a sucrose flush (250 mM) to reduce the signal from extracellular ^23^Na. After each HP experiment, the livers were perfused an additional 15 min with the KH buffer containing 1.5 mM [U-^13^C]lactate and 0.15 mM [U-^13^C]pyruvate before the tissue was freeze-clamped.

### Polarization procedure and real-time ^13^C NMR spectroscopy of perfused liver

[1-^13^C]pyruvate (4 µL) was polarized in a HyperSense polarizer (Oxford Instruments, Oxford, UK) for 2 h with 15 mM OX063 and Gadoteridol ([Gd^3+^] = 2 mM). The polarized sample was rapidly solubilized in a superheated KH buffer (4 mL). Three milliliters of the resultant solution was quickly mixed with 20 mL of KH buffer and injected directly into the perfused liver through a polyethylene catheter. The final concentration of HP [1-^13^C]pyruvate in the injected buffer was 2 mM. No other oxidizable substrates were present in this solution. A series of ^13^C NMR spectra were acquired every 2 s immediately after the injection of HP-pyruvate using 20-deg pulses in a 400 MHz vertical-bore NMR spectrometer (Agilent, Santa Clara, CA) equipped with a 25-mm broadband probe (Doty Scientific, Columbia, SC). The spectra were zero-filled to 64k data points, Fourier-transformed, phased, and baseline-corrected. The calculated areas under the peak were normalized to the total summed peak areas of all ^13^C signals. The signal from pyruvate-hydrate was considered an impurity and excluded from the analysis.

### High-resolution ^1^H and ^13^C NMR spectroscopy of tissue extracts

Frozen liver tissues were pulverized and extracted with 5% perchloric acid. The mixture was neutralized, freeze-dried, and reconstituted in D_2_O containing 0.5 mM DSS (2,2-dimethyl-2-silapentane-5-sulfonate) as an internal standard and 0.1 mM EDTA. ^1^H NMR spectra collected with solvent-presaturation (noesypr1d) and proton-decoupled ^13^C NMR spectra were acquired in a 5 mm NMR tube with the temperature controlled at 37 °C using a 14.1 T Bruker Avance III HD equipped with a 10-mm cryoprobe (Bruker, MA, U.S.A.). The FIDs were zero-filled to 64k data points, Fourier-transformed, phased, and baseline-corrected using ACDLabs SpecManager (ACD/Labs, Canada). All chemical shifts were referenced to DSS set to 0 ppm.

### ^13^C NMR isotopomer assessment of hepatic metabolism

The peak areas of each glutamate C2, C3, and C4 multiplet were measured using ACD software (ACD/Labs, Canada) and used as input for performing a steady-state isotopomer analysis using tcaCALC (download available at http://www.invivometabolism.org/tca.html) as previously described^42,43^. Briefly, the best nonlinear least-squares fit was obtained between calculated and experimental spectra. Fractional multiplet areas measured from the fitting were used for relative flux analyses with a basic TCA cycle model first using the simplest set of metabolic fluxes (i.e. enrichment in acetyl-CoA from the labeled substrates). Additional pathway fluxes such as PC (pyruvate carboxylase, noted as Y_*PC *_in the software) and PyC (pyruvate cycling, noted as PK in the software) were then included in the fitting and the overall agreement between the experimental versus calculated spectral multiplets (Fig. S4) was evaluated. In this model, PyC refers to pathways allowing conversion of a 4-carbon intermediate of the TCA cycle to pyruvate and includes oxaloacetate → phosphoenol pyruvate → pyruvate, and malate → pyruvate. For the ^13^C spectra of liver tissue reported here, the best fit was achieved using a model that included flux of [U-^13^C]pyruvate through PDH (V_*PDH*_), pyruvate carboxylase (V_*PC*_), and pyruvate cycling (V_*PyC*_). The fractional contribution of [U-^13^C]lactate/[U-^13^C]pyruvate to acetyl-CoA was obtained directly from the isotope fitting (there was a small contribution from endogenous sources of acetyl-CoA). Further, tcaCALC data were validated by spectral simulation with tcaSIM (download available at http://www.invivometabolism.org/tca.html). TCA cycle flux (V_*TCA*_) was calculated as reported previously^44,45^. The absolute flux values for PDH flux (V_*PDH*_), pyruvate carboxylase flux (V_*PC*_), and pyruvate cycling flux (*V*_*PyC*_) were obtained by multiplying the relative flux values with V_*TCA*_ (Fig. S2). A ratio of *V*_*PyC*_*/V*_*PC*_ + *V*_*PDH*_ was considered as an estimate of pyruvate cycling. The ratio of lactate ^13^C enrichment due to pyruvate cycling (C2D_23_ + C2D_12_) to total C2 enrichment was also measured for a pyruvate cycling index.

### Histopathology

Liver tissue from each group of animals was trimmed and fixed at 4 °C using 4% formalin/PBS (4% formic acid, 137 mM NaCl, 2.7 mM KCl, and 10 mM phosphate buffer, pH 7.5) and analyzed by the histopathology core of UT Southwestern Medical Center. The tissues were incubated in 10% (w/v) sucrose in PBS for 24 h incubation at 4 °C and transferred to fresh 18% sucrose solution and frozen in optimal cutting temperature compounds (OCT), cryo-sectioned (10 µm), and stained with Oil Red- O (Sigma-Aldrich, USA). Micrographs of tissue sections were taken at a magnification of 20x using an optical microscope (Leica DM2000 Upright Photomicroscope).

### Western blotting

Twenty-five micrograms of protein lysate per lane were used for SDS-PAGE gels. Western blots were transferred to a polyvinylidene difluoride (PVDF) membrane for 2 h at 200 mV and blocked with 5% (wt/vol) nonfat dried milk. The blocked PVDF membrane was probed using polyclonal antibodies to E1α, pE1α, and GAPDH. Antibodies were prepared in the Antibody Core Facility at UT Southwestern Medical Center and affinity purified. Dilution ratios of 1:2 K, 1:25 K, and 1:10 K were used for anti-E1α, anti-pE1α, and secondary IgG detection, respectively. For signal detection, one milliliter of Luminata Forte Western HRP substrate reagent was pipetted across the membrane in a ChemiDoc MP Imaging System (Bio-Rad, USA).

### Statistical analysis

All data are presented as mean ± SD. Statistical analyses were performed using GraphPad Prism. The statistical significance for DKO groups verses their control groups were assessed by an unequal variance (Welch’s) t-test. Multiple statistical comparisons among the weight of livers were calculated using Ordinary one-way ANOVA with ‘Tukey’ post hoc analysis. P values less than 0.05 (*p < 0.05, **p < 0.01 and ***p < 0.001) were considered significant.

All data are presented as mean ± SD. Statistical significance was evaluated by two-tailed unpaired Student’s t-test or one-way ANOVA wherever appropriate using GraphPad Prism (v.7). *P* values less than 0.05 (*p < 0.05, **p < 0.01 and ***p < 0.001) were considered significant.

## Supplementary information

Supplementary Information
