# Supplementary material for: Real-time hyperpolarized 13C magnetic resonance detects increased pyruvate oxidation in pyruvate dehydrogenase kinase 2/4–double knockout mouse livers
Source: Sci Rep. 2019 Nov 11;9:16480. doi: 10.1038/s41598-019-52952-6 (PMC6848094; doi:10.1038/s41598-019-52952-6)
Supplement: Supplementary file 1 — Supplementary Information [file 41598_2019_52952_MOESM1_ESM.docx]

**Supplementary Information**

Real-time hyperpolarized ^13^C magnetic resonance detects increased pyruvate oxidation in pyruvate dehydrogenase kinase 2/4–double knockout mouse livers

**Gaurav Sharma^1^, Cheng-Yang Wu^1^, R. Max Wynn^2,3^, Wenjun Gui^2^, Craig R. Malloy^1,3,4^, A. Dean Sherry^1,4,5^, David T. Chuang^2,3^, and Chalermchai Khemtong^1,4,^***

From the ^1^Advanced Imaging Research Center, University of Texas Southwestern Medical Center, Dallas, TX, United States; ^2^Department of Biochemistry, University of Texas Southwestern Medical Center, Dallas, TX, United States; ^3^Department of Internal Medicine, University of Texas Southwestern Medical Center, Dallas, TX, United States; ^4^Department of Radiology, University of Texas Southwestern Medical Center, Dallas, TX, United States; ^5^Department of Chemistry, University of Texas at Dallas, Dallas, TX, United States.

Running title: ^13^C-NMR detects increased pyruvate oxidation in PDK KO liver

*To whom correspondence should be addressed: Chalermchai Khemtong, University of Texas Southwestern Medical Center, 5323 Harry Hines Boulevard, Dallas, TX 75390-8568, USA. Phone: +1 (214) 645-2772; Email: charlie.khemtong@utsouthwestern.edu


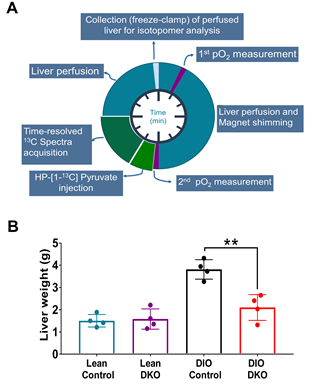


**Figure S1: Perfusion scheme for ^13^C NMR acquisition of hyperpolarized data and liver mass.** (A) Perfusion scheme shows the timeline for ^13^C NMR acquisition of hyperpolarized data. Livers were continuously perfused for 60 minutes and data acquired at the 40th minute of perfusion; (B) Weight of Lean control, Lean DKO, DIO control and DIO DKO perfused mouse livers, note that size of perfused DIO control is significantly higher than other studied groups. Bar plot presented as the mean ± SD (n=4 per group) with significance indicated by “*” (*P* < 0.05) and “**” (*P* < 0.001).


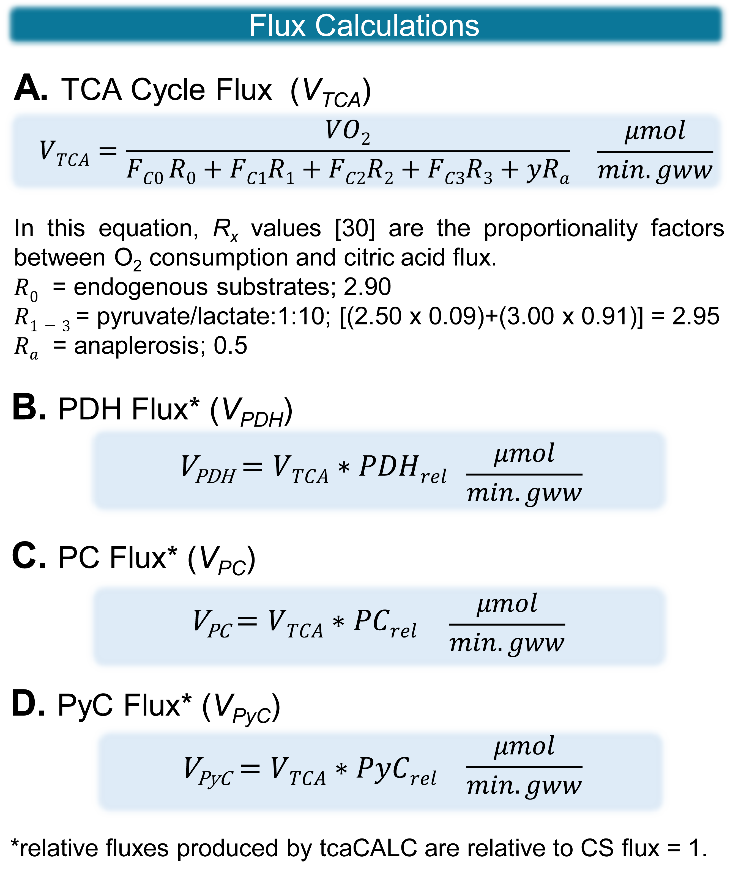


**Figure S2:** **Illustration of** **flux calculations:** Equations for the calculations of (A) TCA cycle flux, (B) PDH flux, (C) PC flux and (D) PyC flux. *V_TCA_*: TCA cycle flux; *PDH_rel_*: Pyruvate dehydrogenase (*V_PDH_*) flux relative to *V_TCA_* , *PC_rel_*: Pyruvate carboxylase (*V_PC_*) flux relative to *V_TCA_* , *PyC_rel_*: Pyruvate cycling (*V_PyC_*) flux relative to *V_TCA_*.


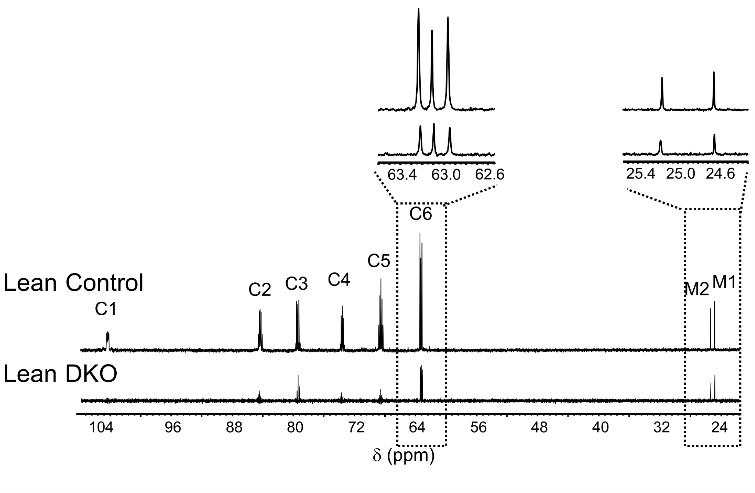


**Figure S3: Impact of DKO on gluconeogenesis:** Representative ^13^C spectra shows the relative intensities of MAG from Lean control (top) and Lean DKO (bottom). The highlighted spectra showed the acetone doublet (M1 and M2) and the C6 multiplet of MAG.


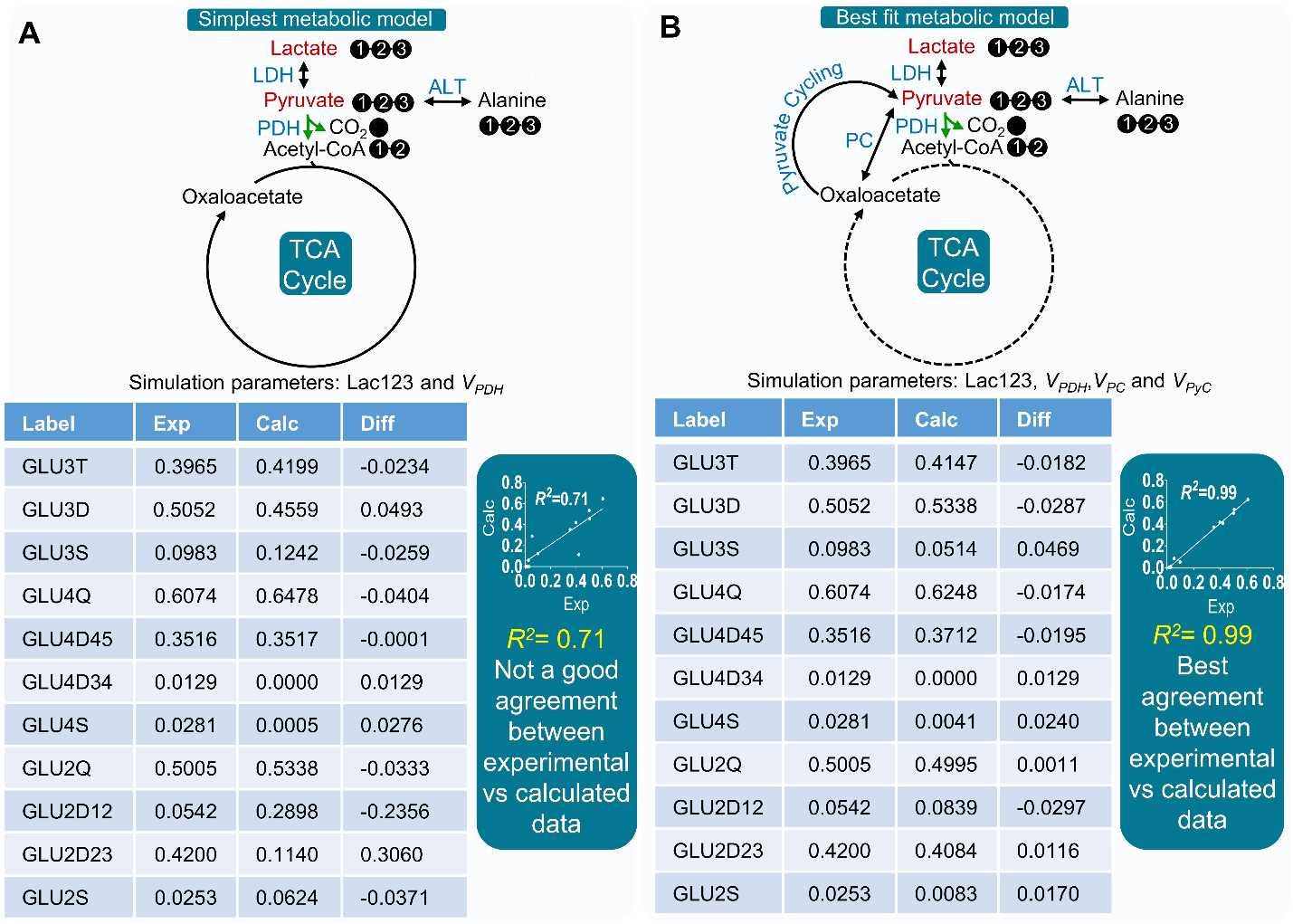


**Figure S4:** **Illustration of** **metabolic modeling with a representative data-set:** Illustration of metabolic modeling and ^13^C NMR isotopomer analysis with an example of NMR data from tissue extract of perfused isolated mouse liver. Calc: Calculated data; Exp: Experimental data; Diff: Difference between experimental and calculated data; *V_PDH_*: Pyruvate dehydrogenase complex flux, *V_PC_*: Pyruvate carboxylase flux; *V_PyC_*: Pyruvate cycling flux, *R^2^*: Coefficient of determination.

**
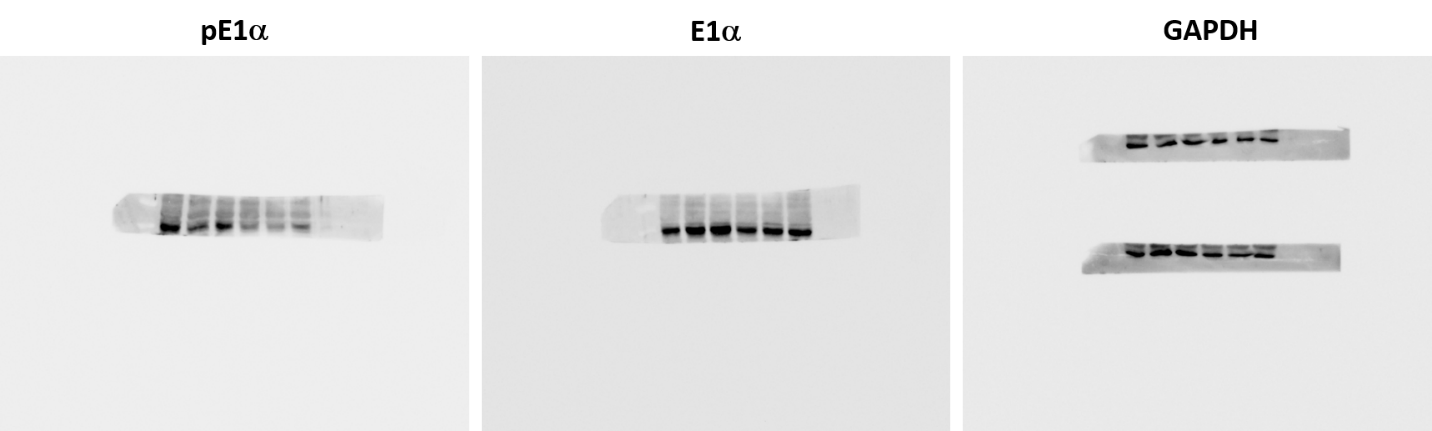
**

**Figure S5:** **Western blots of mouse livers:** Raw images of Western blots shown in Fig. 6, showing protein levels of phosphorylated E1α (left) and total E1α (middle) subunits. Blots (two separate gels) of a loading control GAPDH is shown on the right. The top gel was used in Fig. 6. The images were acquired using a ChemiDoc MP Imaging System (Bio-Rad, USA).
